# Responses of Soil Nitrogen-Cycling Microbial Communities and Functional Potential to Grazing Intensities in Alpine Meadows

**DOI:** 10.3390/microorganisms14051022

**Published:** 2026-04-30

**Authors:** Tianyu Qie, Dong Lin, Qingshan Fan, Guangxu Sun, Hongmei Wang, Zhiyi Liu, Xuepeng Liu

**Affiliations:** 1 Key Laboratory of Grassland Ecosystem (Gansu Agricultural University), Ministry of Education, Lanzhou 730070, China; 15027723895@163.com (T.Q.); fanqs@gsau.edu.cn (Q.F.); 17333885027@163.com (G.S.); 18854054735@163.com (H.W.); 18831086518@163.com (Z.L.); 15002573476@163.com (X.L.); 2 College of Pratacultural Science, Gansu Agricultural University, Lanzhou 730070, China

**Keywords:** alpine meadow soil, grazing intensity gradient, soil microorganisms, metagenomic profiling, grassland restoration

## Abstract

Although grazing is a key driver of nitrogen cycling in alpine meadow soils, a systematic understanding of how different grazing intensities shape the structure and functional potential of soil nitrogen-cycling microbial communities remains lacking. In this study, soil samples were collected under five grazing intensities (no grazing, light grazing, moderate grazing, heavy grazing, and extreme grazing) and metagenomic sequencing was employed to analyze variations in nitrogen-cycling microbial communities and functional genes. The results showed that bacteria were the dominant group in nitrogen-cycling communities (relative abundance: 93.99–98.98%), with significant community differentiation across grazing intensities. Light grazing maintained relatively high microbial diversity, whereas moderate and heavy grazing led to more pronounced differences in community composition. Functional gene analysis identified 41 nitrogen-cycling-related genes, primarily involved in denitrification, nitrate reduction, and ammonia assimilation. Light grazing enhanced nitrate reduction and glutamate synthesis; moderate grazing exhibited the strongest ammonia assimilation potential; heavy grazing significantly increased denitrification activity, indicating an elevated risk of nitrogen loss; and under extreme grazing, both the number and abundance of nitrogen-cycling functional genes declined markedly, with functional composition becoming simplified. Collectively, light grazing is more conducive to maintaining the balance between soil microbial diversity and nitrogen-cycling function in alpine meadows, whereas overgrazing disrupts the equilibrium between microbial communities and nitrogen metabolism. This study provides a microbiological basis for the restoration of degraded alpine meadows and sustainable grazing management.

## 1. Introduction

Alpine meadows represent a critical zonal vegetation type in high-altitude regions, playing a key role in soil and water conservation and grassland-based animal husbandry. Nitrogen is widely recognized as a key nutrient limiting primary productivity in terrestrial ecosystems, including alpine meadows, as its low availability relative to biological demand constrains plant growth and ecosystem functioning [1]. Consequently, the transformation and cycling of nitrogen exert a decisive influence on meadow structure and function. Grazing, recognized as the predominant anthropogenic disturbance in alpine meadows, has been shown to directly or indirectly regulate soil nitrogen cycling through livestock feeding, trampling, and manure and urine return [2]. Under the combined effects of global climate change and grazing activities, nitrogen-cycling processes in alpine ecosystems have undergone significant changes, and the ecological consequences of altered nitrogen availability have become a central concern in recent studies [3].

Soil microorganisms are widely recognized as the core drivers of nitrogen cycling, participating extensively in key processes such as nitrogen fixation, nitrification, denitrification, nitrate reduction, and ammonia assimilation [4,5]. It has been well established that their community composition and functional changes are closely linked to soil nitrogen availability [4,5]. Moreover, grazing intensity exerts selective pressure on microbial communities by altering environmental conditions, including soil physical structure, nutrient inputs, and vegetation cover [6]. International research on the effects of grazing on grassland ecosystem nitrogen cycling has reached several consensus findings. First, grazing intensity is a key determinant of microbial community response patterns. Du et al. [7] found in alpine meadows on the Qinghai–Tibet Plateau that moderate grazing significantly increased soil bacterial abundance and diversity indices, suggesting that moderate disturbance may promote microbial community development. Second, the effect of grazing on microbial diversity is nonlinear. For instance, Zhao et al. [8] showed in the Zoige alpine meadow that grazing significantly affects soil nitrogen mineralization rates and their temperature sensitivity, with response patterns varying distinctly across grazing intensities. Furthermore, grazing stress can reduce the complexity and stability of microbial networks. Consistently, Ma et al. [9] found that alpine meadow degradation which is closely associated with overgrazing, significantly reduced soil microbial biomass and nitrogen mineralization rates. In addition, Sun et al. [10] revealed changes in ammonia-oxidizing and denitrifying microbial communities under different grazing intensities, thereby identifying grazing intensity as a key factor regulating the abundance and composition of functional microorganisms.

Despite these advances, several critical knowledge gaps remain. First, most studies have focused on specific functional groups or individual nitrogen-cycling processes, lacking a systematic understanding of the full nitrogen cycle [8,9,10]. Second, the coupling relationship between microbial community structure and functional gene expression under different grazing intensities remains unclear, particularly regarding the phased reorganization of nitrogen metabolism strategies across the full gradient from light to extreme grazing. Third, the threshold effects of grazing intensity on nitrogen-cycling functional potential and the risk of systemic decline have not been elucidated, and empirical evidence is needed to determine whether extreme grazing leads to the simplification of functional gene networks and the collapse of nitrogen metabolism. Consistently, Zhou’s meta-analysis [11] revealed substantial uncertainty in the effects of grazing on carbon and nitrogen cycling in grassland ecosystems, which is partly attributable to differences in research methods and detection techniques. Most existing studies have employed amplicon sequencing or quantitative PCR, which have inherent limitations in the detection range and quantitative accuracy of functional genes, hindering a comprehensive analysis of changes in nitrogen-cycling functional potential. In addition, traditional research has paid little attention to the coupling between soil environmental factors (e.g., bulk density, microbial biomass phosphorus) and functional genes, or to the differential responses of rhizosphere and non-rhizosphere soils. Metagenomic sequencing can simultaneously obtain community composition and functional gene profiles, thereby overcoming these methodological limitations and providing the technical feasibility to elucidate the assembly patterns and functional thresholds of nitrogen-cycling microbial communities under grazing gradients.

Based on the above knowledge gaps, this study proposed the following hypotheses: (1) Grazing intensity has a nonlinear effect on the diversity of nitrogen-cycling microorganisms, with light grazing maintaining higher diversity (consistent with the “moderate disturbance hypothesis”), whereas extreme grazing leads to a significant decline in diversity and a trend toward stochastic community assembly. (2) Changes in microbial community structure are coupled with functional gene composition: under light grazing, nitrogen retention-related functional genes (e.g., those involved in nitrate reduction and glutamate synthesis) are relatively enriched, whereas under heavy grazing, nitrogen loss-related genes (e.g., those involved in denitrification pathways) increase in abundance, with this transition mediated by environmental factors such as soil bulk density and pH. (3) Extreme grazing leads to a systematic decline in both the number and abundance of nitrogen-cycling functional genes, with functional composition tending to become simplified; specific denitrification pathway genes (e.g., *nirK*) may become dominant markers, indicating a systemic decline in nitrogen-cycling functional potential. Using alpine meadow soils under different grazing intensities (no grazing, light, moderate, heavy, and extreme grazing) and employing metagenomic sequencing combined with redundancy analysis and molecular ecological network perspectives, this study aimed to elucidate the stage-specific response patterns of nitrogen-cycling microbial community structure and functional potential along a grazing intensity gradient, to reveal the coupling relationships among microbial communities, functional genes, and environmental factors, and to provide a microecological basis and threshold references for scientific grazing management, degraded grassland restoration, and nitrogen loss risk control in alpine meadows.

## 2. Materials and Methods

### 2.1. Overview of the Study Area and Sample Collection

The study area is located in Tianzhu Tibetan Autonomous County, Wuwei City, Gansu Province (eastern Qilian Mountains), and has a typical plateau continental climate, with an annual mean temperature ranging from −8 °C to 4 °C and an average elevation of approximately 3100 m. The soil type is subalpine meadow soil. Alpine meadows with consistent vegetation, slope aspect, elevation, and soil type were selected, and five grazing treatments were established: no grazing (CK), light grazing (LG), moderate grazing (MG), heavy grazing (HG), and extreme grazing (EG). The grazing practices for each treatment had been consistently maintained for more than 10 years (Table 1). For each treatment, six independent 100 m × 100 m replicate plots were spatially arranged. In August 2023, 15 soil samples (0–20 cm depth) were randomly collected from each plot using an “S”-shaped sampling pattern and mixed to form one composite soil sample representing the spatial heterogeneity of the plot. A total of 30 soil samples (5 treatments × 6 replicates) were obtained. The samples were passed through a 2 mm sieve to remove plant residues and stones. A portion of each sample was air-dried for the determination of soil physicochemical properties such as pH and total nitrogen. Another portion was placed in centrifuge tubes, stored in foam boxes with dry ice, and sent for metagenomic sequencing. The remaining portion was stored at −80 °C for the determination of soil moisture content and microbial biomass carbon, nitrogen, and phosphorus.

### 2.2. Measurement Content and Methods

#### 2.2.1. Soil Physicochemical Properties

Soil physicochemical parameters were determined, including soil moisture content, bulk density, soil pH, total nitrogen, nitrate nitrogen, ammonium nitrogen, microbial biomass carbon, microbial biomass nitrogen, and microbial biomass phosphorus.

Soil pH was measured by the potentiometric method using a pen-type pH meter (pH-220, Hangzhou Qiwei Instrument Co., Ltd., Hangzhou, China). Soil bulk density was determined using the ring knife method with an intact soil core collected in a stainless steel ring (5 cm inner diameter, 5 cm height), which was then dried to constant weight at 105 °C in an oven (HGZF-II-101-3, Shanghai Yuejin Instrument Co., Ltd., Shanghai, China); bulk density was calculated from the mass of oven-dried soil and the fixed volume of the ring knife. Soil moisture content was determined by the oven-drying method: a certain mass of fresh soil was weighed and dried to constant weight at 105 °C, and gravimetric moisture content was calculated from the mass difference between fresh and oven-dried soil. Total nitrogen was determined by the Kjeldahl method using a Kjeldahl distillation unit for digestion, distillation, and titration.

Soil microbial biomass carbon (MBC), microbial biomass nitrogen (MBN), and microbial biomass phosphorus (MBP) were extracted using the chloroform fumigation-extraction method: MBC and MBN were extracted with a 0.5 mol/L K_2_SO_4_ solution, while MBP was extracted with a 0.5 mol/L NaHCO_3_ solution. Concentrations of MBC and MBN in the extracts were determined using a TOC analyzer (Multi N/C 2100S, Analytik Jena, Jena, Germany), and MBP was measured using an automatic microplate reader (Bio-Tek ELX800, BioTek, Winooski, VT, USA).

Ammonium and nitrate nitrogen were extracted with a 2 mol/L KCl solution, shaken for 1 h on a shaker (YJY-880, Shanghai Hengyue Instrument Co., Ltd., Shanghai, China), filtered, and then analyzed using a continuous flow analyzer (San++Compact, Skalar, Breda, The Netherlands).

#### 2.2.2. Soil Microbial Analysis

(1) Sequencing method
  (a) DNA extraction
  Total genomic DNA of the soil microbial community was extracted using the E.Z.N.A.^®^ Soil DNA Kit (Omega Bio-Tek, Norcross, GA, USA) following the standard protocol. DNA concentration and purity were measured, and DNA integrity was assessed by 1% agarose gel electrophoresis. DNA was fragmented using a Covaris M220 ultrasonicator (Covaris, Woburn, MA, USA), and fragments of approximately 350 bp were selected for paired-end (PE) library construction.
  (b) PE library construction
  The PE library was constructed using the NEXTFLEX Rapid DNA-Seq Kit. (Burleson Rd, Austin, USA) The main steps included: (a) end repair and adapter ligation; (b) magnetic bead purification to remove adapter dimers; (c) PCR amplification to enrich the library; and (d) magnetic bead purification to obtain a high-quality sequencing library.
  (c) Bridge PCR and sequencing
  Metagenomic sequencing was performed on an Illumina NovaSeq™ X Plus platform (Shanghai Majorbio Bio-pharm Technology Co., Ltd., Shanghai, China). The workflow included: (a) immobilization of library molecules on the flow cell via complementary primers; (b) formation of bridge structures by the other ends of the molecules with adjacent primers; (c) bridge PCR amplification to generate high-density DNA clusters; (d) denaturation of double-stranded DNA to single-stranded templates; (e) sequencing by synthesis using modified DNA polymerase and fluorescently labeled dNTPs; (f) fluorescence signal collection to identify base types; (g) chemical cleavage to remove fluorescent groups and restore polymerase activity; and (h) multiple cycles of sequencing to obtain high-quality sequence data.
(2) Analytical methods
  (a) Data quality control
  Raw sequencing data were preprocessed using fastp (v0.20.0), including the removal of adapter sequences from both ends of the reads and filtering out low-quality reads (length < 50 bp or average quality score < 20).
  (b) Assembly and gene prediction
  De novo assembly was performed using MEGAHIT (v1.2.9), and contigs with a length of ≥300 bp were retained. Open reading frames (ORFs) were predicted using Prodigal. Gene sequences with a length of ≥100 bp were selected and translated into amino acid sequences.
  (c) Non-redundant gene set construction
  Gene clustering was performed using CD-HIT (v4.6.1) with the following parameters: sequence identity ≥ 90% and coverage ≥ 90%. The longest gene in each cluster was selected as the representative sequence to construct a non-redundant gene set.
  (d) Gene abundance calculation
  Sequence alignment was performed using SOAPaligner (soap2.21release) with a threshold of 95% identity, and the abundance of each gene was calculated based on the alignment results.
  (e) Species and functional annotation
  a. Taxonomic annotation was performed using Diamond (v2.0.13) for BLASTP alignment (e-value ≤ 1× 10^−5^) against the NR database, and the abundance of each species was calculated by summing the abundances of its associated genes.
  b. KEGG functional annotation was performed by aligning the amino acid sequences of the non-redundant gene set against the KEGG database using Diamond (BLASTP, e-value ≤ 1× 10^−5^). The KO, Pathway, EC, and Module categories were assigned to each gene, and the abundance of each functional category was calculated by summing the abundances of the corresponding genes.

### 2.3. Statistical Analysis

Data were organized using WPS Excel. Alpha diversity indices (richness, evenness, and diversity) were calculated using R (version 3.3.1) and the corresponding algorithms in mothur (v.1.30.2; https://mothur.org/wiki/calculators/, accessed on 5 January 2026). Principal coordinate analysis (PCoA) based on Bray–Curtis distances was performed, and ANOSIM and adonis tests were used to identify significant differences among groups at the species, functional, and gene levels. Linear discriminant analysis (LDA) effect size (LEfSe) was performed with an LDA threshold of 3.5, and only genes with LDA scores ≥ 3.5 were retained. Redundancy analysis (RDA) was performed using the vegan package (version 2.4.3) in R. The Kruskal–Wallis rank-sum test was used to identify significant differences in species abundance among multiple groups.

## 3. Results and Analysis

### 3.1. Composition of Soil Nitrogen-Metabolizing Microbial Communities Under Different Grazing Intensities

**Figure 1:**
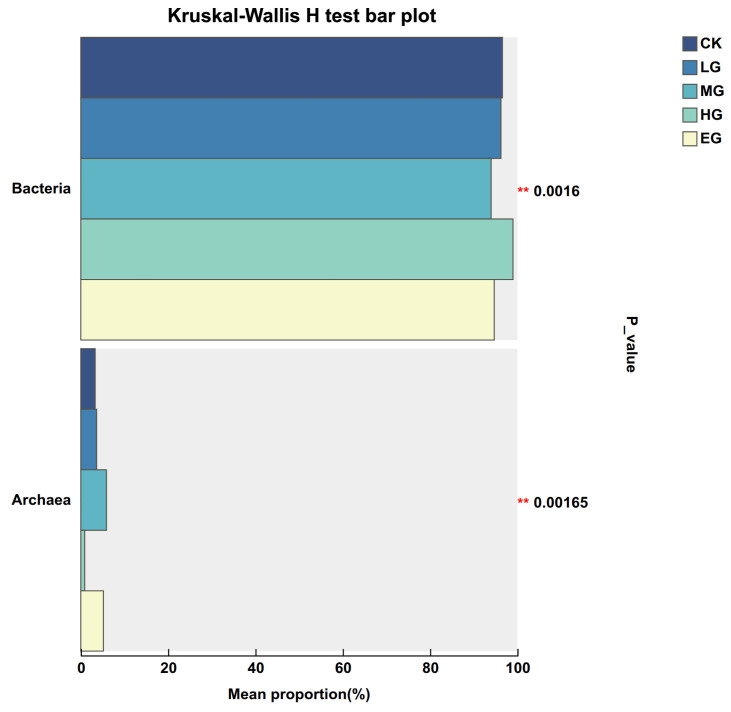
Kruskal–Wallis H-test bar chart of soil nitrogen-metabolizing microbial community composition under different grazing intensities. Note: The vertical axis represents species names at different taxonomic levels, the horizontal axis represents the percentage abundance of a given species in the sample, and different colors represent different groups. ** 0.001 < *p* ≤ 0.01.

Based on annotation against the NR database, a total of 3 domains, 4 kingdoms, 115 phyla, 204 classes, 353 orders, 624 families, 1752 genera, and 5916 species of soil microorganisms involved in nitrogen metabolism were identified in the alpine meadow soils. Bacterial abundance ranged from 93.99% to 98.98%, with the lowest value under moderate grazing and the highest under heavy grazing. Significant differences in bacterial abundance were detected among grazing intensities (*p* < 0.05), and the difference between heavy grazing and moderate grazing was highly significant (*p* < 0.01). Archaeal abundance ranged from 0.89% to 5.86%, with the lowest value under heavy grazing and the highest under moderate grazing. Grazing intensity significantly affected archaeal abundance (*p* < 0.05), with significant differences between moderate grazing and both light and heavy grazing (*p* < 0.01). The relative abundance of eukaryotes ranged from 0.12% to 0.14%, with the lowest value under heavy grazing and a value of 0.14% under all other grazing intensities. No significant difference in eukaryotic abundance was detected among grazing intensities (*p* > 0.05). In summary, bacteria dominated the soil microorganisms involved in nitrogen metabolism in the alpine meadow, with archaea and eukaryotes accounting for smaller proportions (Table 2). Analysis of significant differences between species is shown in Figure 1.

At the order level, the most abundant taxa across all grazing treatments were Hyphomicrobiales and Solirubrobacterales. Their relative abundances varied with grazing intensity, with Hyphomicrobiales peaking under light grazing (18.3%) and Solirubrobacterales under moderate grazing (12.0%) (both *p* < 0.01). Nitrososphaerales showed a non-significant fluctuation (*p* > 0.05) (Supplementary Figures S1a and S2a).

At the species level, *Candidatus Rokubacteria bacterium*, *Luteitalea* sp., and *Nitrososphaeraceae archaeon* all exhibited significant responses to grazing intensity (*p* < 0.01). Detailed taxonomic composition data are provided in Supplementary Materials (Supplementary Figures S1b and S2b).

### 3.2. Changes in the Diversity of Microbial Communities Involved in Nitrogen Metabolism in Soils of Alpine Meadows Under Different Grazing Intensities

**Figure 2:**
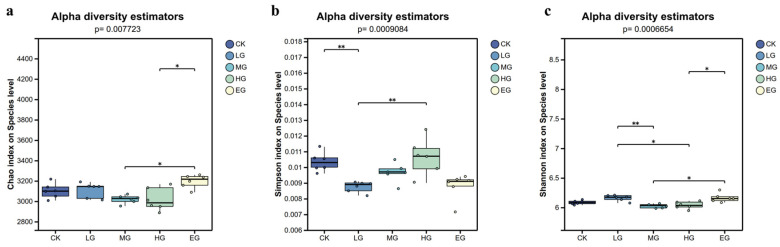
Soil nitrogen-metabolizing microbial community α-diversity. Note: (**a**) Chao index; (**b**) Shannon index; (**c**) Simpson index. * 0.01 < *p* ≤ 0.05; ** 0.001 < *p* ≤ 0.01.

The richness index (Chao index), diversity index (Shannon index), and evenness (Simpson index) of the soil nitrogen-metabolizing microbial communities differed significantly across grazing intensities (Figure 2). The Chao index indicated that species richness in the EG treatment was significantly higher than in the MG and HG treatments (*p* < 0.05). The Shannon index indicated that species diversity in the LG treatment was significantly higher than in the MG and HG treatments (*p* < 0.05) and extremely significantly higher than in the MG treatment (*p* < 0.01), while in the EG treatment it was significantly higher than the HG treatment (*p* < 0.05). The Simpson index indicated that species evenness in the CK treatment was significantly higher than in the LG and MG treatments (*p* < 0.01), and evenness in the HG treatment was relatively high.

**Figure 3:**
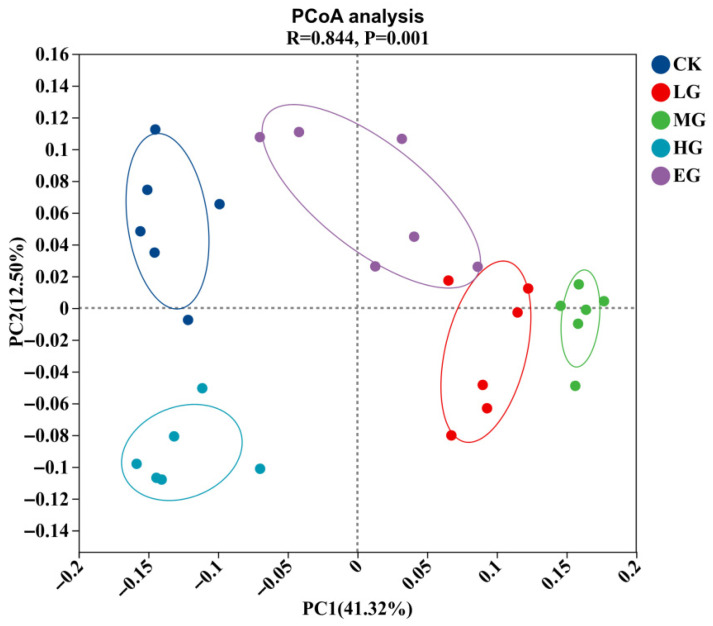
Principal coordinate analysis of soil nitrogen-metabolizing microbial community.

The results of principal coordinate analysis (PCoA) based on Bray–Curtis distance indicate (Figure 3) that there are significant differences in the soil microbial community structure of alpine meadows under different grazing intensities (*p* < 0.01). PC1 and PC2 together explained 53.82% of the variation in community structure, and the intergroup confidence ellipse (*R* = 0.844, *p* = 0.001). Based on the confidence ellipses, the community compositions of the CK and HG treatments were clearly separated from those of the LG, MG, and EG treatments. The community compositions of the LG and EG treatments were relatively similar but still differed. The community composition within the MG treatment showed high homogeneity, while the community dispersion within the EG treatment increased significantly (*p* < 0.01).

### 3.3. Functional Characteristics of Nitrogen-Metabolizing Microbial Communities in Soils of Alpine Meadows Under Different Grazing Intensities

#### 3.3.1. Changes in the Relative Abundance of Genes Involved in Nitrogen Metabolism

**Figure 4:**
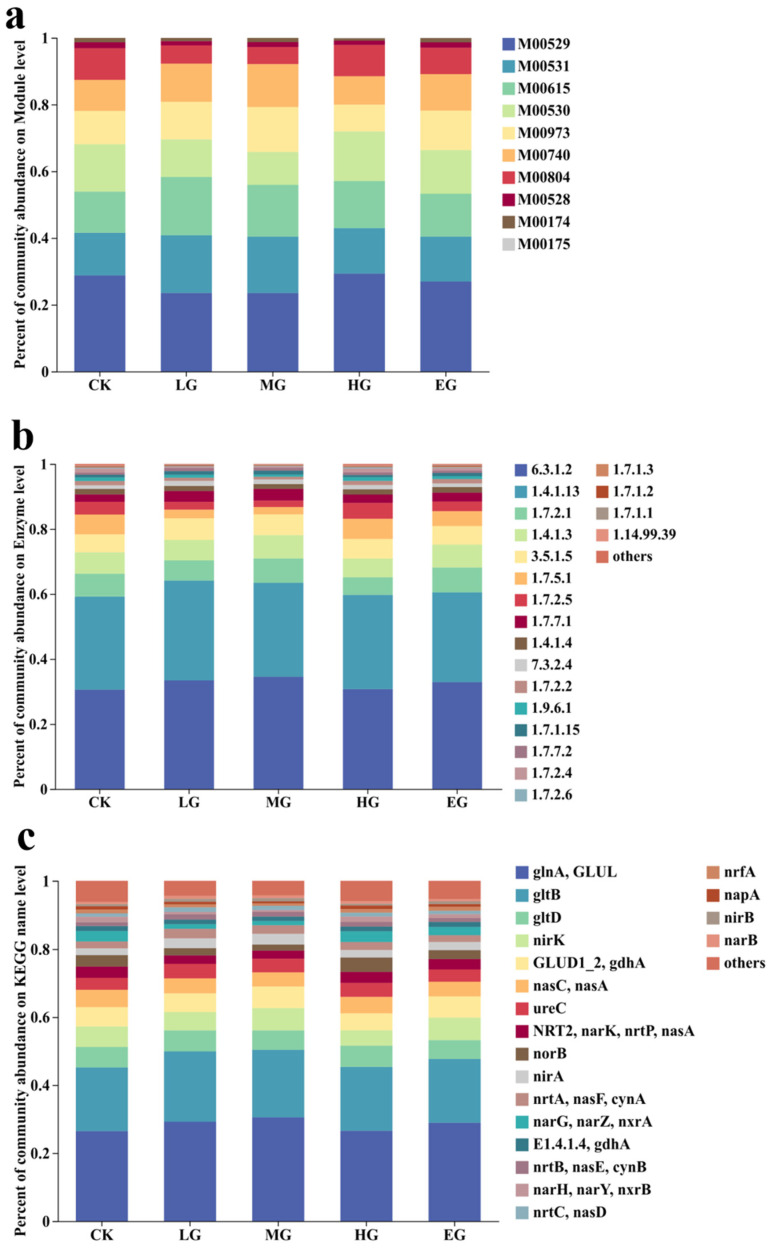
Effects of different grazing intensities on the relative abundance of soil nitrogen metabolism-related functional genes. Note: Top 20 genes by abundance. (**a**) Relative abundance of genes in the nitrogen metabolism module. (**b**) Relative abundance of genes encoding key enzymes in nitrogen metabolism pathways: 6.3.1.2 (glutamine synthetase), 1.4.1.13 (glutamate synthase), nitrite reductase (1.7.2.1, 1.7.7.1, 1.7.2.2, 1.7.1.15), nitrate reductase (1.7.5.1, 1.7.7.2), 1.4.1.3 (glutamate dehydrogenase), 3.5.1.5 (urease), 1.7.2.5 (nitric oxide reductase), 1.4.1.4 (glutamate dehydrogenase), 7.3.2.4 (ATP-binding protein of nitrate/nitrite transport system), 1.9.6.1 (cytochrome-type nitrate reductase), 1.7.2.4 (nitrous oxide reductase), 1.7.2.6 (hydroxylamine dehydrogenase), NAD(P)H-dependent nitrate reductase (1.7.1.3, 1.7.1.2), 1.14.99.39 (methane/ammonia monooxygenase). (**c**) Relative abundance of nitrogen metabolism genes.

After quality control and assembly, the metagenomic data were aligned against the KEGG database, and a total of 41 nitrogen metabolism-related functional genes were identified. Under the different grazing intensities (Figure 4a), the dominant module genes were those involved in denitrification (M00529) and nitrate reduction (M00531, M00615), which together accounted for approximately 60% of the total. The denitrification pathway (M00529) accounted for 23–29% of the total, with the highest proportion under heavy grazing. The nitrate reduction pathways (M00531, M00615) accounted for 12–17% of the total, with the highest proportion under light grazing.

At the enzyme gene level (Figure 4b), glutamine synthetase (6.3.1.2) and glutamate synthase (1.4.1.13) were the most abundant. Glutamine synthetase accounted for 30–34% of the total, with the highest proportion under MG. Glutamate synthase accounted for 27–30% of the total, with the highest proportion under LG. The proportion of nitrate reductase (1.7.5.1) under CK (6%) and HG (6%) was twice that under LG (3%) and three times that under MG (2%); the proportion under EG (4%) was twice that under MG. The proportion of nitric oxide reductase (1.7.2.5) under CK (4%) and HG (4%) was twice that under LG (2%) and MG (2%), with an intermediate proportion under EG (3%).

At the functional gene level (Figure 4c), *glnA/GLUL* (glutamine synthetase) and *gltB* (glutamate synthase) were the most abundant. *glnA/GLUL* accounted for 26–30% of the total, with the highest proportion under MG. *gltB* accounted for 18–20% of the total, with the highest proportion under LG. The proportion of *norB* (denitrification, nitric oxide reductase) showed a decrease–increase–decrease pattern, with the proportion under HG (4%) being twice that under LG (2%) and MG (2%), and the proportion under CK (3%) being equal to that under EG (3%). The proportion of *narG/narZ/nxrA* (complete nitrification, nitrate reductase) also showed a decrease–increase–decrease pattern, with the proportions under CK (3%) and HG (3%) being three times that under LG (1%) and MG (1%), and the proportion under EG (2%) being twice that under LG and MG.

#### 3.3.2. LEfSe Differential Analysis of Genes Related to Nitrogen Metabolism

**Figure 5:**
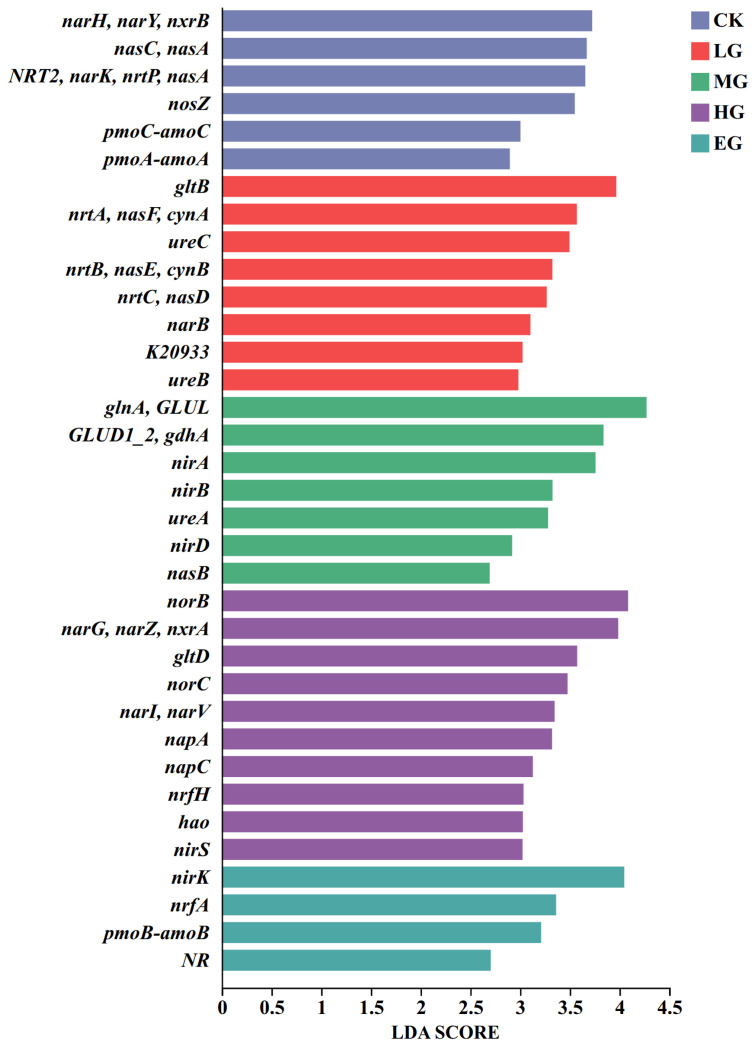
LDA discriminant bar plot of nitrogen metabolism functional genes.

Significant differences were observed in the signature functional genes associated with microbial nitrogen metabolism in alpine meadow soils under different grazing intensities (*p* < 0.05). As shown in Figure 5 and Table 3, under the control (no grazing) conditions, *NRT2/nar*K/*nrt*P/*nas*A (LDA ≈ 3.7, *p* < 0.01) and *nas*C/*nas*A (LDA ≈ 3.7, *p* < 0.01) both exhibited high LDA values and highly significant differences, making them characteristic differential genes. Under light grazing conditions, *ure*C (LDA ≈ 3.5, *p* < 0.01) and *glt*B (LDA ≈ 4.0, *p* < 0.01) both exhibited high LDA values and highly significant differences, making them characteristic differentially expressed genes. Under moderate grazing conditions, *gln*A/*GLUL* (LDA ≈ 4.3, *p* < 0.01) and *GLUD1_2/gdh*A (LDA ≈ 3.8, *p* < 0.01) both exhibited high LDA values and highly significant differences, representing functional genes. Under heavy grazing conditions, only the *glt*D gene exhibited a high LDA value (3.6) and a highly significant difference (*p* < 0.01), making it the characteristic differentially expressed gene for the heavy grazing group; under extreme grazing conditions, the *nir*K gene served as the characteristic differentially expressed gene, with an LDA value of 4.0 and *p* < 0.01.

### 3.4. Correlation Between Soil Environmental Factors and Microbial Community Structure Under Different Grazing Intensities

**Figure 6:**
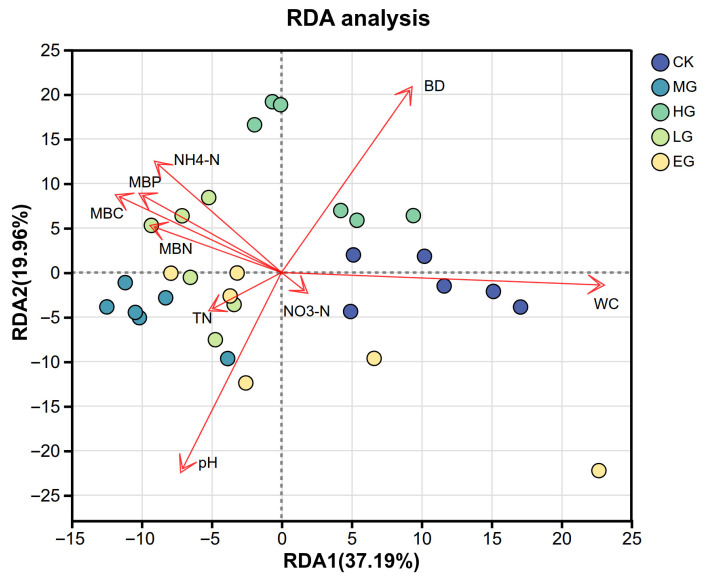
Redundancy analysis (RDA) of environmental factors and soil nitrogen-metabolizing microbial community structure under different grazing intensities.

Redundancy analysis (RDA) was performed to explore the relationships between the soil nitrogen-metabolizing microbial community structure and environmental factors under different grazing intensities (Figure 6). The results showed that RDA1 and RDA2 explained 37.19% and 19.96% of the total variation in the community, respectively, with a cumulative explanation of 57.15%, indicating that the environmental factors effectively explained the community differentiation. The samples from different grazing treatments were clearly separated along the RDA1 axis: the CK and HG samples clustered on the right side of the axis, while the LG, MG, and EG samples clustered on the left side, indicating that grazing intensity significantly altered the microbial community structure. Among the environmental factors, soil pH, microbial biomass carbon (MBC), microbial biomass phosphorus (MBP), soil bulk density (BD), and ammonium nitrogen (NH_4_^+^-N) were the core factors driving community differentiation. MBC, MBP, MBN, NH_4_^+^-N, and total nitrogen (TN) were positively correlated with the LG and MG groups; BD was positively correlated with the HG group; pH was positively correlated with the LG, MG, and EG groups but negatively correlated with the CK and HG groups; soil water content (WC) was positively correlated with the CK and EG groups; and NO_3_^−^-N was positively correlated with the CK group.

## 4. Discussion

This study systematically investigated the structure, diversity, functional gene composition, and environmental coupling of soil microbial nitrogen-cycling communities in an alpine meadow under different grazing intensities (no grazing, light grazing, moderate grazing, heavy grazing, and extreme grazing). The key findings were as follows: (1) Bacteria dominated the nitrogen-cycling microbial communities, and their abundance differed significantly among grazing intensities. (2) Grazing intensity had a nonlinear effect on microbial diversity: light grazing maintained relatively high diversity, whereas extreme grazing led to community simplification. (3) A total of 41 nitrogen-cycling functional genes were identified, primarily involved in denitrification, nitrate reduction, and ammonia assimilation, and their relative abundances varied with grazing intensity in a stage-specific manner. (4) Light grazing enhanced nitrogen retention functions; moderate grazing showed the strongest ammonia assimilation potential; heavy grazing significantly activated denitrification; and under extreme grazing, both the number and abundance of functional genes declined systematically. (5) Environmental factors significantly drove community differentiation. These results strongly support the hypothesis that grazing intensity influences the assembly and functional potential of nitrogen-cycling microbial communities by regulating the soil microenvironment and resource availability.

### 4.1. Nonlinear Regulation of Nitrogen-Cycling Microbial Community Structure and Diversity by Grazing Intensity

In this study, bacteria remained the dominant group across all grazing intensities (relative abundance: 93.99–98.98%), reflecting their strong adaptation to the cold, anaerobic, and organic-rich conditions of the alpine meadow soil. Bacterial abundance peaked under heavy grazing (98.98%) and reached its lowest level under moderate grazing (93.99%), whereas archaea showed the opposite trend (highest under moderate grazing, lowest under heavy grazing), indicating that grazing intensity differentially regulates these two major prokaryotic groups. This finding is consistent with that reported by Yi et al. [12] in alpine meadows on the Qinghai–Tibet Plateau, who found that grazing disturbance significantly alters the competitive balance between bacteria and archaea, thereby affecting nitrogen transformation efficiency.

The response of microbial diversity to grazing intensity was nonlinear. The Shannon index peaked under light grazing, consistent with the “moderate disturbance hypothesis.” Du et al. [7] found in alpine meadows on the Qinghai–Tibet Plateau that moderate grazing significantly increased soil bacterial abundance and diversity indices, suggesting that moderate disturbance may promote microbial community development. Consistently, Kang et al. [13] reached a similar conclusion in a metagenomic study of permafrost alpine meadows on the Qinghai–Tibet Plateau, showing that moderate disturbance maintains high microbial diversity, whereas excessive disturbance leads to community simplification. Furthermore, Zhao et al. [8] reported that grazing significantly affects soil nitrogen mineralization rates and their temperature sensitivity in the Zoige alpine meadow, with distinct response patterns across grazing intensities, thereby further supporting the nonlinear regulation of microbial communities by grazing intensity.

Notably, Zhu et al. [14] found in a 17-year grazing experiment in a desert steppe in Inner Mongolia that light grazing (0.91 sheep∙hm^−2^) partially maintained microbial nitrogen transformation potential, whereas heavy grazing led to a marked decline in functional gene abundance. They also showed that the rhizosphere soil microbial community responded more sensitively to grazing than did the non-rhizosphere community, providing an important direction for future research.

PCoA analysis revealed significant differences in community structure among grazing intensities (*p* < 0.01), with the CK and HG groups clearly separated from the other groups along the first axis. Molecular ecological network analysis has elucidated the impact of grazing on microbial interactions. Further evidence comes from Sun et al. [15], who found in Tibetan grasslands that grazing significantly altered interactions among carbon- and nitrogen-cycling functional genes. Notably, both carbon- and nitrogen-cycling gene networks exhibited scale-free and small-world properties under both control and grazing conditions. Moreover aboveground plant biomass was significantly correlated with the topological characteristics of the microbial gene network (*p* = 0.001), confirming the close aboveground–belowground coupling. In parallel, Ma et al. [16] reported that alpine meadow degradation which is closely associated with overgrazing, significantly reduced soil microbial biomass and nitrogen mineralization rates. In the present study, the within-group dispersion of the EG group was significantly increased, which may reflect increased fragmentation of microbial interaction networks and increased stochasticity of community assembly under extreme grazing.

### 4.2. Stage-Specific Reorganization of Nitrogen-Cycling Functional Genes Driven by Grazing Intensity

A total of 41 nitrogen-cycling functional genes were identified, primarily involved in denitrification, nitrate reduction, and ammonia assimilation. The composition and relative abundance of these genes showed distinct stage-specific patterns with increasing grazing intensity, reflecting adaptive adjustments in nitrogen metabolism strategies. This finding is consistent with the meta-analysis by Yin et al. [17], who systematically reviewed 83 studies and identified grazing intensity as a key factor regulating the abundance of nitrogen-cycling functional genes.

Light grazing enhanced nitrogen retention and nitrate reduction. Under LG, the nitrate reduction pathways (M00531, M00615) and the glutamate synthase gene (*gltB*) had the highest relative abundances, and LEfSe identified *gltB* and *ureC* as signature genes of the LG group. This agrees with the study by Sui et al. [18] in a spring alpine meadow, which showed that light grazing significantly increases soil nitrogen availability, with the core mechanism involving the activation of nitrate reduction and ammonia assimilation. Zhu et al. [14] similarly observed in a desert steppe that nitrogen-cycling functional genes had higher relative abundances under light grazing than under moderate or heavy grazing, and structural equation modeling revealed that the increase in soil pH was significantly associated with microbial community changes and a decrease in the net nitrogen mineralization rate. This cross-ecosystem consistency suggests that the maintenance of nitrogen retention by light grazing may have general ecological relevance.

Moderate grazing showed the strongest ammonia assimilation potential. Under MG, the glutamine synthetase gene (*glnA/GLUL*) and the glutamate dehydrogenase gene (*GLUD1_2/gdhA*) had the highest relative abundances, and both were signature genes of the MG group. In addition, archaeal abundance was significantly higher under MG (5.86%) than under the other treatments, and the abundance of Nitrososphaerales reached 4%, four times that of CK. Archaea are key drivers of ammonia oxidation, and their increased abundance may form a positive feedback loop with the increased supply of the ammonia assimilation substrate (NH_4_^+^). Ma et al. [16] further showed that different dominant plant communities in alpine meadows regulate the abundance and composition of ammonia-oxidizing microorganisms, thereby affecting soil nitrification rates and N_2_O emissions, Together, these findings provide a new perspective for understanding the cascading effects of grazing–vegetation–microbe interactions.

Pan et al. [19] provided important supporting evidence in a study of an alpine meadow in the Qilian Mountains. They found that moderately disturbed winter grazing helps maintain the structural and functional stability of the microbial community in alpine grasslands, whereas fencing and monoculture of oats significantly alter the fungal community structure. They also showed that grassland management practices change the distribution of potassium (K) by altering the aboveground plant community structure (grasses and legumes) and belowground biomass allocation, leading to significant changes in nitrogen metabolism enzymes. These findings suggest that the enhancement of ammonia assimilation under moderate grazing may be achieved through a “vegetation–nutrient–microorganism” cascade pathway.

Heavy grazing significantly activated denitrification, increasing the risk of gaseous nitrogen loss. Under HG, the denitrification pathway module gene (M00529) had the highest relative abundance (29%), and the abundance of *norB* (4%) was twice that under LG and MG. LEfSe showed that the number of genes with high LDA values but non-significant differences increased in the HG group, with only *gltD* remaining significantly different. Consistently, Sun et al. [10] found that heavy grazing significantly increases the abundance of denitrifying microorganisms and the activity of denitrification-related genes, leading to increased N_2_O emissions. Mechanistically, in the present study, soil bulk density (BD) in the HG group was significantly positively correlated with community structure (RDA), suggesting that soil compaction due to livestock trampling may create anaerobic microsites that promote denitrification.

The meta-analysis by Yin et al. [17] provided global comparative data: moderate-to-heavy grazing reduced N_2_O emissions by 22–25%, decreased nitrification rates by 23–37%, decreased denitrification rates by 44–48%, reduced AOB *amoA* abundance by 40–47%, and reduced *narG* and *nirS* abundances by 28% and 35%, respectively. At first glance these findings appear to contrast with the denitrification gene enrichment observed under HG in the present study. However, it should be noted that the meta-analysis focused on changes in N_2_O flux, whereas the present study measured functional gene abundance; importantly, an increase in gene abundance does not necessarily translate into an increase in flux, as there may be post-transcriptional or post-translational regulation at the transcriptional or enzymatic level. Furthermore, Yin et al. [17] explicitly indicated that the effect of grazing on N_2_O emissions is indirect, mediated by changes in soil water content and inorganic nitrogen availability.

Tang et al. [20] conducted a warming–grazing interaction experiment in an alpine meadow on the Qinghai–Tibet Plateau and found that grazing alone significantly increased the α-diversity of functional genes, altered the overall functional community structure, and increased the abundance of genes involved in carbon fixation, carbon degradation, nitrogen mineralization, and denitrification, possibly due to the stimulatory effect of excreta deposition. They also found an antagonistic interaction between warming and grazing, suggesting that the effect of grazing on nitrogen cycling may be partially offset under future climate warming.

Under extreme grazing, the number and abundance of nitrogen-cycling functional genes declined systematically. Under EG, the total number of nitrogen metabolism-related functional genes decreased, and the abundances of all major pathway genes were lower than those under LG and MG. LEfSe identified *nirK* as the only signature gene of the EG group, indicating selective activation of the nitrite reduction step in denitrification but a general decline in overall nitrogen transformation capacity. This result is consistent with the study by Zhang et al. [21], who found that extreme environmental disturbances lead to simplified functional gene differentiation and the collapse of nitrogen metabolism networks. Zhang et al. [21] studied grasslands along a drought gradient on the Tibetan Plateau and identified an aridity threshold (aridity index >0.6) above which the rate of decline in soil multifunctionality slowed with increasing aridity, but the abundances of denitrification genes (*nosZ*, *norB*) increased significantly. This suggests that extreme grazing may push nitrogen-cycling functions from a “diversity-driven” mode to a simplified “stress-resistant-gene-dominated” mode via a stress threshold mechanism.

Relevant insights also emerge from Huang et al. [22], who found in degraded alpine meadows that artificial restoration measures (revegetation) induced a “significant increase–stability–increase” trend in the diversity of carbon-cycling functional genes. Notably, the top 15 dominant microbial species contributed > 40% of the carbon-cycling functional genes. Together, these findings suggest that for severely degraded areas, assisted restoration (e.g., reseeding, organic fertilizer application) may be necessary to restore nitrogen-cycling functions.

### 4.3. Coupling Between Environmental Factors and Microbial Communities Drives Functional Differentiation of Nitrogen Metabolism

Redundancy analysis (RDA) showed that soil pH, microbial biomass carbon (MBC), microbial biomass phosphorus (MBP), soil bulk density (BD), and ammonium nitrogen (NH_4_^+^-N) were the core environmental factors driving the differentiation of nitrogen-cycling microbial communities. MBC, MBP, MBN, NH_4_^+^-N, and total nitrogen (TN) were significantly positively correlated with the LG and MG groups, indicating that moderate grazing increases carbon and nitrogen inputs, thereby promoting microbial growth and nitrogen retention. BD was significantly positively correlated with the HG group, confirming the selective effect of trampling compaction on community structure.

The structural equation model of Zhu et al. [14] similarly showed that the increase in soil pH under long-term grazing was significantly associated with changes in the microbial community and a decrease in the net nitrogen mineralization rate. A study on spring rest–grazing timing [23] identified soil temperature and water infiltration rate as key environmental variables regulating functional gene composition, corroborating the finding that environmental factors dominate community differentiation in the present study.

Notably, eukaryotic abundance did not differ significantly among grazing intensities (*p* > 0.05), indicating that grazing primarily affects bacterial and archaeal communities and has a limited effect on the participation of eukaryotic microorganisms (e.g., fungi) in nitrogen metabolism. This finding is consistent with the “aboveground–belowground linkages” theory of Bardgett and Wardle [6], which states that the effect of grazing on the soil food web is trophic-level-specific, with the bacterial channel being more sensitive to grazing disturbance than the fungal channel.

### 4.4. Implications for Grazing Management and Restoration of Degraded Alpine Meadows

The stage-specific responses observed in this study indicate that light grazing (2.24 sheep∙hm^−2^∙yr^−1^) is more conducive to maintaining the balance between soil microbial diversity and nitrogen-cycling functions in the alpine meadow. At this intensity, nitrate reduction and ammonia assimilation are enhanced, denitrification losses are suppressed, and nitrogen retention efficiency is relatively high. In contrast, heavy-to-extreme grazing (≥5.24 sheep∙hm^−2^∙yr^−1^) leads to denitrification dominance, functional gene simplification, and reduced community stability, significantly increasing the risk of nitrogen loss.

Pan et al. [19] explicitly recommended that moderate disturbance (e.g., winter grazing) is beneficial for maintaining microbial community stability in alpine grasslands. Methodologically, the meta-analysis by Zhou [11] indicated that the effects of grazing on carbon and nitrogen cycling in grassland ecosystems are highly uncertain, partly because of differences in research methods and detection techniques, thereby highlighting the importance of metagenomic approaches for comprehensively assessing nitrogen-cycling functional potential. From a management perspective, the meta-analysis by Yin et al. [17] cautioned that although moderate-to-heavy grazing can reduce N_2_O emissions, overgrazing may increase the risk of grassland degradation, and grazing intensity should therefore be controlled at a moderate level to balance production and emission reduction.

Therefore, when formulating grazing management strategies for alpine meadows, the grazing intensity should be controlled within the light-to-moderate threshold range (approximately 2–4 sheep∙hm^−2^∙yr^−1^), and long-term heavy grazing should be avoided. For severely degraded areas that have experienced extreme grazing, a combination of short-term grazing exclusion and organic amendments (e.g., composted sheep manure) is recommended to restore soil carbon and nitrogen resources and microbial functional networks. This recommendation is supported by Huang et al. [22], who showed that artificial revegetation can effectively restore soil functional gene diversity, thereby providing a feasible pathway for the restoration of severely degraded ecosystems.

### 4.5. Limitations and Future Directions

Although this study used metagenomic sequencing to comprehensively map the nitrogen-cycling functional gene profile, several limitations should be acknowledged. First, functional gene abundance reflects potential rather than actual metabolic rates. Future studies should combine ^15^N tracing or N_2_O flux measurements to validate the quantitative relationship between functional gene expression and nitrogen loss risk. Second, the study used a space-for-time substitution and lacked long-term temporal dynamics along a grazing gradient; long-term monitoring platforms should be established. Third, Tang et al. [20] showed an antagonistic interaction between warming and grazing; future studies should track the effects of grazing under climate change scenarios. Fourth, the present study did not distinguish between rhizosphere and non-rhizosphere soils, but Zhu et al. [14] showed that the rhizosphere microbial community responds more sensitively to grazing stress, representing an important direction for future research. Fifth, the study did not consider the network interactions of functional genes. Sun et al. [15] suggested that network analysis could help to understand the maintenance mechanisms of microbial functional stability under grazing stress.

Despite these limitations, this study systematically reveals, for the first time, the stage-specific response patterns of soil nitrogen-cycling microbial communities and functional potential along a grazing intensity gradient from light to extreme in an alpine meadow. The findings provide empirical evidence and threshold references based on microbial ecology for grazing management and the restoration of degraded grasslands.

## 5. Conclusions

This study systematically revealed the nonlinear response patterns of soil nitrogen-cycling microbial communities under different grazing intensities in alpine meadows. Bacteria were the dominant group; light grazing maintained the highest microbial diversity, while extreme grazing led to community simplification. A total of 41 nitrogen-cycling functional genes were identified, which exhibited stage-specific changes with increasing grazing intensity. Light grazing favored nitrogen retention, moderate grazing resulted in the strongest ammonia assimilation, heavy grazing significantly promoted denitrification, and extreme grazing caused an overall decline in functional genes. Soil environmental factors were the core drivers of community differentiation. The results indicate that light-to-moderate grazing helps maintain the balance between soil microbial diversity and nitrogen-cycling functions, whereas heavy-to-extreme grazing increases the risk of nitrogen loss. These findings provide a microbial ecological basis for grazing management and the restoration of degraded alpine meadows.

## Figures and Tables

**Table 1:** Overview of sampling areas.

Grazing Intensity	Altitude (m)	Cover Degree (%)	Grazing Rate (sheep·hm^−2^·a^−1^)	The Main Plant
No grazing (CK)	3107	100	0	*Elymus nutans*, *Bistorta vivipara*, *Kobresia pygmaea*, *Ranunculus japonicus*, *Poa annua*, *Dasiphora fruticosa*, *Potentilla anserina*
Light grazing (LG)	3047	95	2.24	*Kobresia pygmaea*, *Bistorta vivipara*, *Elymus nutans*, *Artemisia smithii*, *Melilotoides ruthenica*, *Kobresia capillifolia*, *Gentiana macrophylla*
Moderate grazing (MG)	2894	90	4.15	*Kobresia pygmaea*, *Melilotoides ruthenica*, *Elymus nutans*, *Artemisia smithii*, *Leontopodium nanum*, *Stipa capillata*
Heavy grazing (HG)	3424	85	5.24	*Potentilla strigosa*, *Kobresia pygmaea*, *Bistorta vivipara*, *Dasiphora fruticosa*, *Elymus nutans*
Extreme grazing (EG)	3110	60	7.94	*Kobresia pygmaea*, *Melilotoides ruthenica*, *Leymus secalinus*, *Potentilla strigosa*

Note: Conversion grazing rate = sheep unit number/(grazing time × grazing area). One white yak = 5 sheep units. One Tianhua Merino sheep = 1 sheep unit. One Gansu fine wool sheep = 1 sheep unit (according to NY/T-635-2015 national standard).

**Table 2:** Distribution of soil nitrogen-metabolizing microorganisms.

Grazing Intensity	Bacteria (%)	Archaea (%)	Eukaryotes (%)
No grazing (CK)	96.58	3.27	0.14
Light grazing (LG)	96.2	3.64	0.14
Moderate grazing (MG)	93.99	5.86	0.14
Heavy grazing (HG)	98.98	0.89	0.12
Extreme grazing (EG)	94.78	5.07	0.14

**Table 3:** LDA discrimination results table.

Gene Name	Grazing Intensity	LDA Value	*p* Value
*NarH/narY/nxrB*	CK	4.05	*p* > 0.05
*NRT2/narK/nrtP/nasA*	CK	3.57	*p* < 0.01
*NasC/nasA*	CK	3.73	*p* < 0.01
*nrtA/nasF/cynA*	LG	3.50	*p* > 0.05
*ureC*	LG	3.99	*p* < 0.01
*gltB*	LG	4.09	*p* < 0.01
*glnA/GLUL*	MG	3.48	*p* < 0.01
*nirA*	MG	3.57	*p* > 0.05
*GLUD1_2/gdhA*	MG	3.66	*p* < 0.01
*NarG/narZ/nxrA*	HG	4.27	*p* > 0.05
*norB*	HG	3.97	*p* > 0.05
*norC*	HG	3.76	*p* > 0.05
*gltD*	HG	3.84	*p* < 0.01
*nirK*	EG	3.67	*p* < 0.01

Note: Genes with LDA values ≥3.5 are listed for each grazing intensity. Only genes with *p* < 0.05 were considered statistically significant.

## Data Availability

The original contributions presented in this study are included in the article/Supplementary Material. Further inquiries can be directed to the corresponding author.

## Supplementary Materials

The following supporting information can be downloaded at https://www.mdpi.com/article/10.3390/microorganisms14051022/s1, Figure S1: Community composition bar chart. Figure S2: Kruskal–Wallis H-test bar charts of soil nitrogen-metabolizing microbial community composition under different grazing intensities.
