# Supplementary material for: Responses of Soil Nitrogen-Cycling Microbial Communities and Functional Potential to Grazing Intensities in Alpine Meadows
Source: Microorganisms. 2026 Apr 30;14(5):1022. doi: 10.3390/microorganisms14051022 (PMC13209816; doi:10.3390/microorganisms14051022)
Supplement: Supplementary file 1 [file microorganisms-14-01022-s001.zip › microorganisms-4245313-supplementary.pdf]

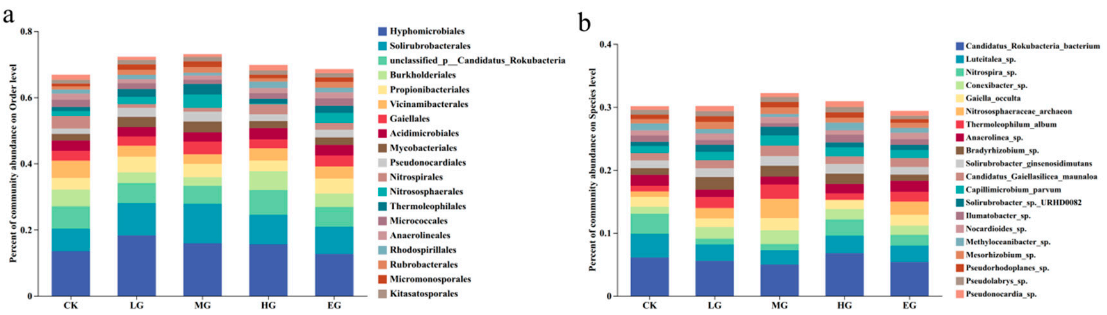

Figure S1. Community composition bar chart.

Note: (a) shows a bar chart of community composition at the order level; (b) shows a bar chart of community composition at the species level; The figure shows the top 20 species by abundance, with low-abundance species grouped under “others.” The x-axis represents different grazing intensity groups: no grazing (CK), light grazing (LG), moderate grazing (MG), heavy grazing (HG), and extreme grazing (EG). The y-axis represents relative abundance. The differently colored blocks on the right side of the figure represent the names of different microbial species. The same applies below.

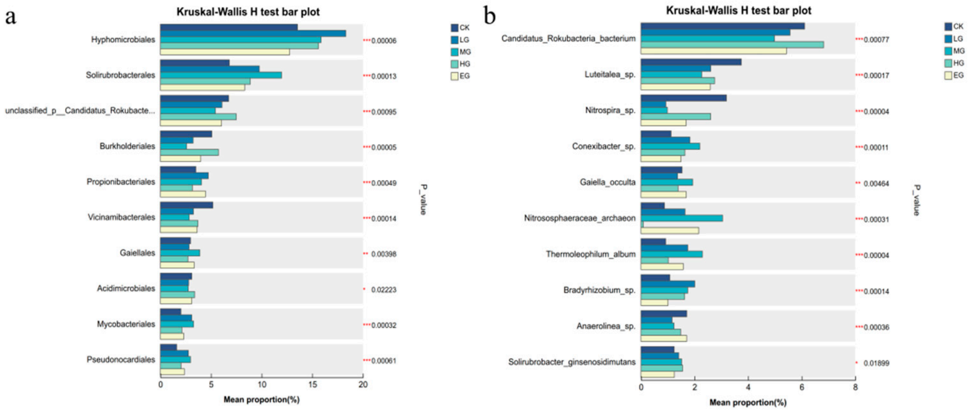

Figure S2. Kruskal–Wallis H-test bar charts of soil nitrogen-metabolizing microbial community composition under different grazing intensities.
